# Design of a Participatory Organizational-Level Work Stress Prevention Approach in Primary Education

**DOI:** 10.3389/fpsyg.2022.827278

**Published:** 2022-03-30

**Authors:** Maartje C. Bakhuys Roozeboom, Irene M. W. Niks, Roosmarijn M. C. Schelvis, Noortje M. Wiezer, Cécile R. L. Boot

**Affiliations:** ^1^TNO (The Netherlands Organization for Applied Scientific Research), Leiden, Netherlands; ^2^Department of Public and Occupational Health, Amsterdam Public Health Research Institute, VU University, Amsterdam, Netherlands; ^3^Department of Public and Occupational Health, Coronel Institute of Occupational Health, Amsterdam Public Health Research Institute, Amsterdam UMC, University of Amsterdam, Academic Medical Center, Amsterdam, Netherlands

**Keywords:** intervention research, logic model, implementation, participatory organizational level work stress prevention approach, primary education

## Abstract

**Background:**

Work stress is a serious problem in primary education. Decades of research underline the importance of participatory, organizational-level work stress prevention approaches. In this approach, measures are planned to tackle causes of work stress in a participatory manner and implemented by a working group consisting of members of the organization. This approach can only be effective if the measures contain effective ingredients to decrease work stress risks and are successfully implemented. The aim of this paper is to present an outline of a work stress prevention approach that is evaluated in primary education. To ensure the appropriateness of measures, a logic model of change is built as part of the risk assessment to facilitate the selection of appropriate measures. Progression on target behaviors as well as implementation factors are real-time monitored during implementation and fed back to the working groups, to provide the opportunity to adjust action plans when needed to optimize implementation.

**Methods:**

The approach consists of five steps: (1) *preparation*: installing an advisory board and working groups, (2) *risk assessment*: inventory of work stress risks (questionnaires and focus groups). In addition, a behavioral analysis is performed to build a logic model of change to facilitate selection of measures, (3) *action planning*: conducting an action plan with appropriate measures (focus groups), (4) *implementation*: implementing the action plan. During implementation progression on target behaviors and implementation factors are monthly monitored and fed back to the working groups, and (5) *evaluation*: effects of the approach are studied in a controlled trial with measurements at baseline (T0), 1 year (T1), and 2 years (T2) follow-up. A process evaluation is carried out using quantitative (questionnaires and real-time monitoring data) and qualitative (interviews and data logs) data to study the implementation process of all steps of the work stress approach.

**Discussion:**

We believe that building a logic model of change and real-time monitoring of implementation could be of added value to improve the success of the work stress prevention approach. With this study, we aim to provide more insights into work stress intervention research, especially in primary education.

**Clinical Trial Registration:**

The study is registered in Netherlands Trial Register (ClinicalTrials.gov #NL9797, October 18, 2021).

## Introduction

### Work Stress in Primary Education

Work stress is a serious problem among workers throughout the globe. Especially, workers in primary education are at risk to suffer from work stress. Data from Netherlands Working Condition Survey (Hooftman et al., 2021) show that the highest proportion of employees that report work stress are employed in the educational sector. From research, it is known that work stress can have severe consequences for workers’ health by causing cardiovascular diseases (Kivimäki et al., 2012), musculoskeletal disorders (Da Costa and Vieira, 2010), and mental health problems (Harvey et al., 2017). Work stress among teachers can also have negative consequences for schools, leading to decreased commitment (Klassen et al., 2013) and increased sickness absence (Duijts et al., 2007) and also for students, leading to low quality of education (Varghese and Kurian, 2020). In addition, there is a substantial shortage of teachers in primary education in Netherlands and high levels of work stress make working in this sector less appealing, and may also increase the risk of turnover (Weiss, 1999; Perrachione et al., 2008). These results underline the urgency to combat work stress in education.

### Causes of Work Stress

Several theoretical models describe the potential causes of work stress (e.g., Job Demands Control [Support]-model [JDC(S)model; Karasek, 1979], the Demand-Induced Strain Compensation-model [DISC-model; De Jonge and Dormann, 2003], and Job Demands Resources-model [JDR-model; Bakker and Demerouti, 2007]). These models focus on a balance principle: work stress is caused by an imbalance between high job demands and low resources. Job demands are organizational, social, and physical aspects of the job that require effort (Schaufeli et al., 2009). Resources refer to aspects of the job that reduce job demands, help achieve work goals, and stimulate personal development (Demerouti et al., 2001). Resources can be divided into organizational resources (e.g., supervisor support, co-worker support, and autonomy) and personal resources (e.g., resilience and optimism). Research on teachers’ causes of work stress identified several specific job demands and organizational and personal resources that are related to (1) the workload, e.g., time pressure, difficult students, being confronted with continuous change, and administrative tasks (Kyriacou, 2001; Hakanen et al., 2006; Roeser et al., 2013; Fitchett et al., 2016; McCarthy, 2016), (2) social interrelations, e.g., lack of social support from colleagues or management (Kyriacou, 2001; Roeser et al., 2013), and (3) personal characteristics, e.g., coping mechanism (Kyriacou, 2001).

### Participatory, Organizational-Level Stepwise Approach for Work Stress Prevention

Given the previously mentioned scarcity of teachers, the high prevalence of work stress, and the severe consequences, there is a need for effective work stress interventions in education. However, research shows that work stress interventions in primary education are lacking or not effective. International meta-analyses showed only limited, low-quality studies (Naghieh et al., 2015) or small effects (Iancu et al., 2018). Most of the studied interventions aimed at teachers’ work stress or burnout are person-directed interventions that target secondary risk prevention (e.g., relaxation training, mindfulness, and cognitive behavioral theory; von der Embse et al., 2019). However, scholars question whether these types of interventions are the most sustainable approach to work stress prevention (Lamontagne et al., 2007). According to the “hierarchy of control” principle, interventions are most (cost)effective if they target work stress risks at their source (e.g., job demands and resources).

An approach in this respect that received an increasing interest in the past decades is the participatory, organizational-level stepwise work stress prevention approach (Kompier and Marcelissen, 1995; Cox et al., 2003; Nielsen et al., 2010a; Leka et al., 2011). In this approach, actions are planned to remove or modify causes of work stress in a participatory manner and implemented by a working group consisting of workers and management from the organization (implementors). In general, the approach consists of five steps: (1) *preparation*: preparation and planning of the practical aspects of the approach, (2) *risk assessment*: inventory of work stress risks, (3) *action planning*: planning measures to target risks, (4) *implementation*: implementing measures by means of an action plan, and (5) *evaluation*: evaluation of the approach.

Although these organizational-level approaches hold the potential to sustainably reduce work stress since they target work stress risks at their source, in practice these interventions often fail to bring about the expected outcomes (Semmer, 2003). There can be several explanations for this: the selected measures do not consist of the *effective ingredients* to decrease causes of work stress (measures are not appropriate; Nielsen et al., 2010b), the measures are not *implemented successfully* (Nielsen et al., 2010a), or a combination of both factors. In this paper, we outline the planning of a work stress prevention approach that is implemented and evaluated in primary education. To diminish the risks mentioned above (not selecting appropriate measures and/or implementation failure), for this study, the work stress prevention approach is expanded with (1) building a logic model of change to facilitate action planning and (2) real-time monitoring of the implementation process.

### Logic Model of Change

When it comes to the underlying mechanism of work stress, reducing job demands and increasing organizational and personal resources often requires behavioral actions of different actors within the organization. Examples of such behaviors are as: managers prioritizing work tasks, managers providing feedback to employees, employees taking work breaks, etc.

Traditional risk assessments often focus on common risk factors as described in dominant work stress theories (Nielsen et al., 2010b). These risk assessments may, for example, reveal that a particular department suffers from work stress due to high job demands. However, they often do not specify what kind of behavioral change is needed from whom to reduce these job demands. Making more explicit what behavioral change the measures should aim for would facilitate the selection of measures and secure that measures contain appropriate and effective behavioral change methods.

Frameworks for the development of behavioral change interventions that are well adopted in the general health domain, stress the importance of conducting a logic model of change to better define the active ingredients of measures that are needed to accomplish the intended outcomes [e.g., Intervention Mapping (Bartholomew Eldredge et al., 2016)]. This requires a behavioral analysis to (1) formulate the program objective and performance objectives (specific behavioral actions needed to reach the program objective), (2) identify determinants for each performance objective, and (3) propose theory-based intervention methods that target the determinants and help achieve the performance objectives. The result of this behavioral analysis is a logic model of change, which represents pathways of the work stress prevention approach’s effects, and points out the behavioral changes necessary to achieve the intended health outcome (reduce work stress). Building this logic model of change could be of added value to the work stress prevention approach because it provides guidance for selecting and planning appropriate measures that contain effective behavior change methods.

### Real-Time Monitoring Implementation

Even when appropriate measures are planned, they need to be successfully implemented to accomplish the intended effects. As Nielsen et al. (2010a) pointed out in practice the implementation of work stress prevention approaches often is hindered by factors related to the implementation process. Implementation factors that are considered important for successful implementation are management commitment, participation of employees or support from employees, tailored and timely communication, and/or mental models of the workers (readiness for change; Nielsen et al., 2010a).

In their study, Lien and Saksvik (2016) monitored attitudes toward organizational change through monthly assessments and results were communicated to the change managers via feedback loops during the organizational change. This approach of real-time monitoring during the implementation process and providing feedback to implementors holds potential to reduce the risk of implementation failure. Monitoring important implementation factors (management commitment, employee participation, communication, and readiness for change) during implementation and providing feedback to implementors may stimulate implementors to take behavioral actions the moment when hindrances are identified. This may reduce the risk of implementation failure.

In a similar manner, monitoring progress on outcomes (work stress), risk factors, and target behaviors and providing feedback to implementors provides the opportunity to adjust and optimize measures when needed. According to the Goal Setting Theory (Latham and Locke, 1975; Locke and Latham, 2019), monitoring and receiving feedback on the progression of goals appears to be positively related to goal pursuit. In addition, this type of feedback could provide more guidance to adjust action plans during implementation by changing existing measures or introducing new ones when needed.

Another advantage of real-time monitoring during the implementation process is that data on the implementation process are collected as the implementation evolves. A weakness of most process evaluations is that the evaluation often takes place after the implementation of the intervention (retrospective; Nielsen et al., 2010a). This can challenge identification of implementation hindrances due to recall bias. Data collected with real-time monitoring of the implementation process may facilitate the process evaluation, by providing a picture on changes in implementation factors over time.

### Aim of This Paper

To summarize, the aim of this paper is to present an outline of a work stress prevention approach that is evaluated in primary education. To ensure the appropriateness of measures, a logic model of change is built as part of the risk assessment to facilitate the selection of appropriate measures. During implementation, progression on outcomes, risk factors, and target behaviors as well as implementation factors are real-time monitored and fed back to the working groups, to provide the opportunity to adjust action plans when needed and reduce the risk of implementation failure.

## Methods and Analysis

**Figure 1:**
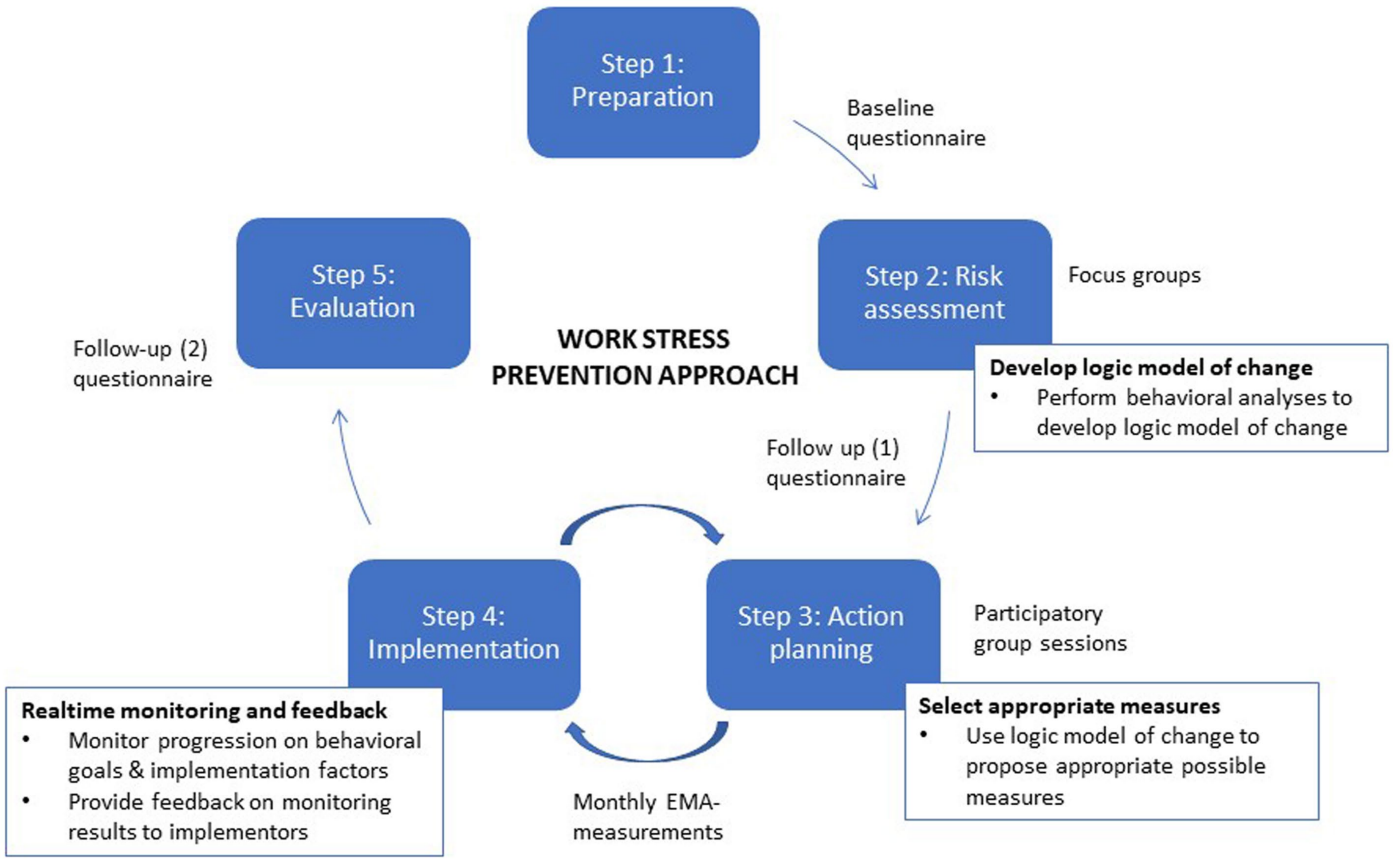
Schematic overview of work stress prevention approach.

This paper outlines a work stress prevention approach that will be conducted in primary education in Netherlands and evaluated in a controlled trial. The approach consists of five steps: (1) preparation, (2) risk assessment, (3) action planning, (4) implementation, and (5) evaluation (see Figure 1). As part of the risk assessment (step 2), a behavioral analysis is carried out to build a logic model of change. This logic model is used to select measures for action planning (step 3). During implementation (step 4), progression on target behavior and implementation factors are monitored and feedback is provided to implementors. After implementation, the effect of the approach and the implementation process are evaluated (step 5).

Following the conceptual model of participation in work environment interventions (Abildgaard et al., 2020), the work stress prevention approach is participatory in the sense that during risk assessment, action planning and implementation employees have direct and indirect (via working group) influence over the focus, content and implementation of the intervention activities.

### Study Population

The study population (intervention and control group) consists of teaching and non-teaching staff (i.e., managers and support staff) from 30 schools in primary education (*N* = 739) that fall under the scope of two school cooperations. The schools differ in size and include small, medium, and large schools, and are located in the middle of Netherlands.

All schools received an invitation to participate in the intervention group. Of both school cooperations a large and a small school that were willing to participate were appointed as intervention schools (*N* = 102). These four schools follow the five steps of the work stress prevention approach. All other 26 schools (*N* = 637) are appointed as control schools and only take part in the questionnaire measurements at baseline, 1, and 2 years follow-up (see Figure 1).

### Step 1 Preparation

The study protocol is tested and approved by an ethical committee. All employees receive information about the study and sign an informed consent for the study activities. An advisory board to the intervention project is installed that consists of the management of the school cooperation and members of the research team. Regular meetings are planned with the advisory board to discuss preliminary results and progression of the project. In addition, regular meetings are planned between the research team and the principal of the intervention schools to discuss progress, preliminary results, and collect feedback. At each of the four intervention schools, a working group is installed consisting of 2–3 employees and the school principal. The working group is responsible to conduct and implement a school-specific action plan.

### Step 2 Risk Assessment

The risk assessment is aimed at the identification of causes of work stress for teachers in primary education. As part of the risk assessment, focus group meetings are carried out (two focus group meetings with 3–5 employees per school). In the focus group meetings, participants are asked to think of factors that cause, contribute to, and buffer work stress and to think about the interrelations between these factors. Post-its are used to organize factors into one schematic model that reflects the interrelations of risk factors and the dynamic nature of work stress development for the participants. The results of the different workshops are combined into one schematic model that covers all factors that are mentioned in the workshops. This model is used by the researchers to identify the most important risk factors to reduce work stress among workers in primary education by selecting factors that are often mentioned in the different workshops, and factors that are related to many other factors.

### Behavioral Analysis

**Figure 2:**
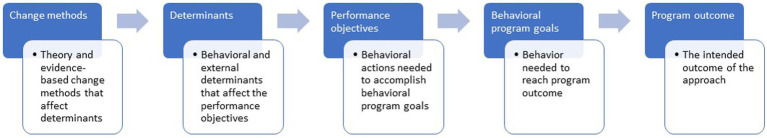
Steps of the behavioral analysis resulting in a logic model of change.

Based on the identified risk factors, a behavioral analysis is carried out by the research team to develop a logic model of change that reflects the situation for all four schools (see Figure 2). First, the intended outcome of the intervention is formulated (e.g., work stress reduction among primary school workers). Second, this intended outcome is translated in terms of behavior by determining what behavior is needed to prevent work stress among workers in primary education (e.g., keep a healthy energy balance, carry out work tasks within regular working hours). These are the behavioral program goals the measures (that are selected in step 3 action planning) should focus on. Third, behavioral actions (performance objectives) that are needed from different actors to accomplish the behavioral program goals are specified (e.g., monitor workload and exchange expectations with colleagues). Fourth, behavioral and external determinants of these behavioral actions are identified that are a precondition for the behavioral actions to occur (e.g., motivation, self-efficacy, and awareness). Last, the research team selects suitable theory- and evidence-based change methods (e.g., guided practice and goal setting) aimed at the identified determinant, based on behavioral change literature (Bartholomew Eldredge et al., 2016). The advisory board and the school principals are consulted to check the (preliminary) results of the behavioral analyses (e.g., do they reflect practice?).

### Step 3 Action Planning

As part of the action planning (Step 3), possible work stress measures are inventoried by means of participatory focus group sessions with employees. At each school, sessions are organized with all employees to collect and discuss possible measures that match with the needs based on the risk assessment and that fit the context of the school. This inventory of measures combined with the results of the behavioral analysis are used by the research team to make one general action plan including a logic model of change. This general action plan includes several appropriate possible measures and the rationale behind these measures (logic model of change). This general action plan is handed over to the working groups at the schools.

At each school, a kick-off meeting is organized with the working group to select and specify measures from the general action plan into a school-specific action plan. The action planning and implementation of measures follow an iterative action approach, meaning that the school-specific action plans are constantly evolving during the implementation period, measures can be changed, and new measures can be introduced overtime, until an optimum is reached.

### Step 4 Implementation

The working groups at the schools are responsible for the implementation of measures of the school-specific action plans. During implementation, the working groups regularly meet and discuss progression of the action plan and make changes if needed. The frequency of meetings is decided upon by the working group members based on their needs and preferences. When needed, schools can get in contact with the other intervention schools to learn from each other’s experiences (buddy system).

### Real-Time Monitoring

The working groups receive feedback from monitoring data, collected by monthly Ecological Momentary Assessment (EMA) measurements among all employees of their schools. EMA involves repeated sampling of subjects’ current feelings, states, behaviors, and experiences, in real-time and in subjects’ natural environment (Shiffman et al., 2008; Kirchner and Shiffman, 2013). Results of the monthly EMA measurements at school level are fed back to the working groups to reflect on the progression on work stress, risk factors, target behaviors, and on the implementation process and take behavioral actions if needed. During the implementation period with a duration of 10 months (excluding 2 months of summer holidays), all employees receive 8 short surveys that they can fill in with an app they need to install on their mobile phones (the EMA measurements). Within the app, participants can view a graph with their individual work stress level overtime, based on the monthly measurements.

Results are presented to the working groups in a monthly report that contains graphs of the mean scores of all items at school level. With every new EMA measurement, a new report is conducted with additional scores added to the existing graphs. This way the report presents an overview of trends over time. In addition to the graphs with the mean scores, the report contains reflection questions for the working groups to answer, to reflect on the effectiveness of measures and the implementation process. Examples of reflection questions are as: *“Are there any changes on [work stress risk/ behavioral goals/implementation factors] as compared to last months’ measurement?,” “Are changes on [work stress risk/behavioral goals/implementation factors] in the expected direction?,” “Are additional/other measures needed?,” and “Are additional actions needed to optimize implementation*?.” To reduce the risk of loss to follow-up, the monthly surveys are as short as possible and provide participants with feedback on their work stress levels within the app. This could work as an incentive to participate.

#### Measurements Real-Time Monitoring (EMA)

Items are selected that are deemed relevant to monitor work stress, work stress risks, progression on target behavior, and implementation factors. The number of items is limited to reduce the risk of response loss and minimize the efforts asked of participants. The following items are included in the monthly EMA-measurements.

##### Work Stress

*Work stress* is measured by single item stress question (SISQ; Arapovic-Johansson et al., 2017; *Stress is a state where you feel tense, restless, nervous, anxious, or cannot sleep at night because you are worried. Have you experienced this type of stress in the past month?*). Response scales range from 1 = *low stress* to 100 = *high stress*.

##### Work Stress Risks

*Work stress risks* are measured by single item questions that are selected based on the outcomes of the risk assessment (e.g., *administrative tasks, difficult students, and high expectations from colleagues*). Response scales range from 1 = *not at all* to 10 = *to a very large extent*.

##### Target Behavior

*Target behaviors* are measured by single item questions that are selected based on the behavioral analyses (*To what extent did this statement apply to you considering the last month?* e.g., *I was working on personal goals, I was prioritizing my work tasks*). Response scales range from 1 = *not at all* to 10 = *to a very large extent*.

##### Implementation Factors

*Communication* is measured by two items “I am aware of the objectives of [the project]” and “I am informed about the progress of [the project].” *Commitment* is measured by three items based on the IPM-Q (Randall et al., 2009) “*I have the feeling that the team is positive about [the project],”* “*I have the feeling that the our principal is positive about [the project],”* and “*I have the feeling that the school cooperation is positive about [the project].*” *Participation* is measured by two items based on the IPM-Q (Randall et al., 2009) *“I am involved in [the project]” and “I can think along with the measures that are taken as part of [the project].*” *Readiness for change* is measured by three items based on the Organizational Change Questionnaire–Climate of Change, Processes, and Readiness (OCQ–C, P, R) (Bouckenooghe et al., 2009): “*I am willing to actively contribute to [the project]* (intentional readiness for change), *“I expect that [the project] will help to reduce my work stress”* (cognitive readiness for change), and “*I have a positive feeling about [the project]”* (emotional readiness for change). Response scales range from 1 = *not at all* to 10 = *to a very large extent*.

### Step 5 Evaluation

**Figure 3:**
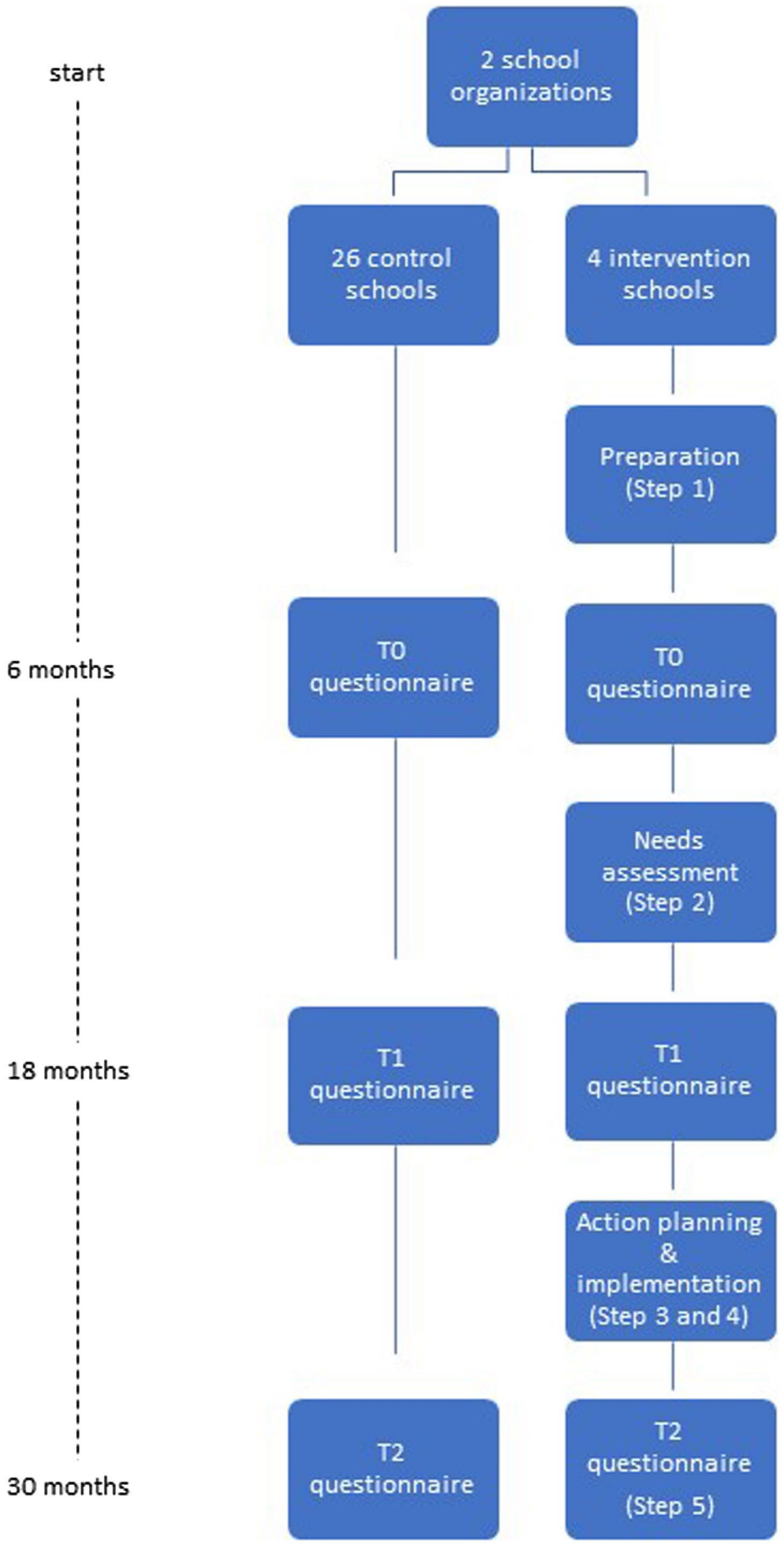
Schematic overview of study design for effect evaluation.

The effects of the work stress prevention approach are evaluated in a controlled trial (see Figure 3). As part of the effect evaluation online questionnaires are sent out at baseline (after preparation), 1 year (after needs assessment), and 2 years follow-up (after implementation) to all workers of the intervention and control schools.

#### Measurements Baseline, One Year, and Two Years Follow-Up Questionnaires

Since the baseline questionnaire (T0) is sent out to the respondents before the logic model of change is developed, we aim to measure several potential job demands and organizational and personal resources that are known to contribute to work stress. This way, we optimize the possibilities to include moderating and mediating factors (based on the logic model of change) when performing the analyses for effect evaluation.

##### Work Stress

*Work stress* was measured with 5 items of the Utrecht Burnout Scale (UBOS; Schaufeli and van Dierendonck, 2000), a slightly adjusted Dutch version of the Maslach Burnout Inventory-General Survey (MBI-GS; Maslach et al., 1986). The selected subset of items primarily measures the emotional exhaustion component of burnout complaints (e.g., *I feel emotionally exhausted by my work*). Response scales range from 0 = never to 6 = every day.

*Self-reported stress* is measured by 2 items based on the Danish Psychosocial Work Environment Questionnaire (DPQ; Clausen et al., 2019; e.g., *How often have you felt stressed within that last 2 weeks?*). Response scales range from 1 = *all the time* to 5 = *never*.

##### Job Demands

*Quantitative demands* are measured by 3 items based on the Dutch version of the Job Content Questionnaire (JCQ; Karasek, 1979, 1985; e.g., *Do you have a lot of work to do?*). Response scales range from 1 = *never* to 4 = *always*.

*Emotional demands* are measured by 3 items based on the Copenhagen Psychosocial Questionnaire (Kristensen et al., 2005; e.g., *Does your work put you in emotionally disturbing situations?*). Response scales range from 1 = *never* to 4 = *always*.

*Unnecessary work tasks* are measured by 4 items based on the Danish Psychosocial Work Environment Questionnaire (DPQ; Clausen et al., 2019; e.g., *Do you spend time on work tasks that you have difficulty seeing the purpose of*). Response scales range from 1 = *to a very large extent* to 5 = *to a very small extent*.

*Time pressure* is measured by 3 items based on the Copenhagen Psychosocial Questionnaire (Kristensen et al., 2005; e.g., *Is it necessary to keep working at a high pace?*). Response scales range from 1 = *never* to 5 = *always*.

*Technostress* is measured by 5 items based on the instrument on Techno-stressors (Ragu-Nathan et al., 2008; e.g., *Due to the increased technological complexity I have a higher workload*). Response scales range from 1 = *Totally disagree* to 5 = *Totally agree*.

##### Organizational Resources

*Autonomy* is measured by 4 items based on the Dutch version of the Job Content Questionnaire (JCQ; Karasek, 1979, 1985; e.g., *Can you decide for yourself how you do your work?*). Response scales range from 1 = *yes regularly* to 3 = *no*.

##### Personal Factors/Resources

*Basic Needs Satisfaction at Work* is measured by a selection of 6 items based on the Basic Needs Satisfaction at Work scale (Kasser et al., 1992; Ilardi et al., 1993; Deci et al., 2001) that measures three dimensions *competence* (2 items, e.g., *I do not feel very competent when I am at work*), *autonomy* (2 items, e.g., *When I am at work, I have to do what I am told*), and *belonging* (2 items, e.g., *There are not many people at work that I am close to*). Response scales range from 1 = *Not at all true* to 7 = *Very true*.

*Self-efficacy about functioning under stress (stress resistance) and recovery after stress (resilience)* are measured by 6 items selected from the Connor-Davidson Resilience Scale (CD-RISC; Connor and Davidson, 2003), Brief Resilience Scale (BRS; Smith et al., 2008), and Mental Toughness Scale (Clough et al., 2002). *Stress resistance* is measured by 3 items (e.g., *Even when I’m under a lot of pressure, I stay calm*). Response scales range from 1 = *Totally disagree* to 5 = *Totally agree. Recovery after stress* is measured by 3 items (e.g., *I recover quickly from setbacks*).

*Optimism* is measured by 3 items based on the Life Orientation Test (Scheier and Carver, 1985; Scheier et al., 1994; e.g., *I’m optimistic about my future*). Response scales range from 1 = *Totally disagree* to 5 = *Totally agree*.

Job crafting is measured by 6 items selected from the Job Crafting Survey (JCS; Tims et al., 2012; e.g., *I make sure that I make optimal use of my capacities*). Response scales range from 1 = *Totally disagree* to 5 = *Totally agree*.

#### Analyses

To study the effect of the work stress prevention approach, per protocol analyses will be performed on the data of the 30 participating primary schools. To adjust for clustering of schools multilevel mixed model analyses are performed. The data from the current study contain three levels; the first level of the data contains the individual scores of the participants on the determinants and outcome (within-subjects level), the second level of the data contains the schools in which the individual participants are nested (between-schools level), and the third level of the data contains the school cooperations in which the schools are nested (between-school cooperation level). However, we expect that differences at this third level are limited, and due to the variety of schools, any clustering will manifest itself at school level. Adjustments for each level are considered and evaluated at the start of the analysis. Multivariate analyses are carried out (for each of the primary and secondary outcomes) with the difference scores of the primary and secondary outcomes as dependent variable, and the centered score of this variable at baseline and condition (intervention versus control) as independent variables. To obtain the amount of variance explained by the differences between the schools, the intraclass correlation coefficient (ICC) is calculated for each analysis. For all hypotheses, a value of *p* < 0.05 is indicated as statistically significant.

#### Power Analysis

The power calculation is based on the sample size needed for the effect evaluation of the work stress prevention approach, including two groups, the intervention schools and control schools with, respectively, 4 and 26 clusters (schools). The estimated average cluster size (considering loss to follow-up) is 15 participants (intervention schools: *N* = 60; control schools *N* = 390). Assuming a significance level (α) of 0.05, two-sided tests and power (1-β) of 0.80 and an ICC for schools of 0.01, we will be able to detect an effect of Cohen’s *d* = 0.43. In their review on burnout prevention programs Awa et al. (2010) found effect sizes between *d* = 0.29 and *d* = 1.2. Note that we did not consider the ICC for school cooperation in the power calculation, because we expect that adjustments for this level are not necessary.

### Process Evaluation

In addition to the effect evaluation, a process evaluation is conducted according to the model for evaluating organizational-level interventions of Nielsen and Randall (2013). The process evaluation in this study uses quantitative data collected with the T0, T1, and T2 questionnaires as well as monthly EMA measurements. In addition, qualitative data are collected by means of interviews and data logs (see Table 1 and paragraphs Interviews and Data Logs).

**Table 1:** Process factors and type of data collection.

Process factor	Research question	Questionnaire (employee)	EMA-measurement (employee)	Interviews (school principal and member working group)	Data logs by research team
Intervention design and implementation					
Initiation	Who initiated the intervention and for what purpose?				X
Developing intervention activities	Did the intervention activities target the problems of the workplace?			X	
Implementing intervention activities (exposure to components of the intervention)	Did the intervention reach the target group?	T2		X	X
Implementation strategy					
Drivers of change and the roles of key stakeholders	Who were/are the drivers of change?			X	X
Employee involvement	Did employees participate significantly in decision making and how many were involved?	T2	X	X	
Management support/commitment	What was the role of senior/middle managers?	T2	X	X	
Information and communication	What kind of information was provided to participants during the study?	T2	X		
Context					
Omnibus context	How did the intervention fit in with the culture and conditions of the intervention group?			X	
Discrete context	Which events took place during the intervention phase?			X	X
Mental models					
Readiness for change	To what extent are/were participants ready for change?	T0	X		
Shared mental models	To what degree do participants have shared mental models?	T0	X		
Appraisal of the intervention and its activities (e.g., satisfaction)	How did participants perceive the intervention and its activities? To what extent are participants satisfied with the intervention?	T2		X	
Changes in mental models	Did the intervention bring about a change in participants’ mental models?	T0, T1, T2	X		

#### Questionnaires

The following items were included in the T0, T1, and T2 questionnaire:

The concept of *Mental models* is measured by 2 items: “*Work pressure of employees at our school is a problem that should be addressed*” and “*I am confident that [the project] will bring me something.”*

The following items were included in the T2 questionnaire:

*Exposure to components of the intervention* is measured by 1 item based on the IPM-Q (Randall et al., 2009): “*I have noticed that measures and/or changes have been implemented as a consequence of [the project].”*

*Communication* is measured by 2 exploratory items: “*I am aware of the objectives of [the project]”* and “*I was informed about the progress of [the project].*”

*Commitment* is measured by 3 items based on the IPM-Q (Randall et al., 2009): “*I have the feeling that the team is positive about [the project],”* “*I have the feeling that the head of our school is positive about [the project],”* and “*I have the feeling that the school organization is positive about [the project].*”

*Participation* is measured by 2 items based on the IPM-Q (Randall et al., 2009) *“I have been involved in [the project]” and “I could think along with the measures that are taken as part of [the project].*”

*Satisfaction* is measured by 1 exploratory item: “*To what extent are you satisfied with [the project]”?*

#### EMA Measurements

Data on the implementation factors that are measured by the EMA measurements as part of the real-time monitoring are used to evaluate changes in readiness for change, communication, commitment, and participation. Items that are included in the EMA measurements are described earlier [see paragraph real-time monitoring (EMA)].

#### Interviews

To collect additional data on the intervention design and implementation, the implementation strategy, the context, and mental models, two interviews are conducted per intervention school: one interview with the school principal and one interview with an employee. Interviews are conducted according to a semi-structured interview protocol, by telephone (*n* = 8) and will last between 30 and 60 min. Minutes are made by a research assistant and interview transcripts are coded according to the following topics: intervention design and implementation, implementation strategy, context, and mental models.

#### Data Logs

During the work stress approach, data are logged by the research team regarding the initiation of the approach and the exposure to components of the intervention (e.g., number of participants taking part in interviews and EMA measurements, division of roles within the schools). In addition, the division of roles is logged within the schools and based on regular contacts with the working group and schools principals, major events during implementation are logged.

## Discussion

This paper outlines the design of an organizational-level participatory work stress prevention approach that will be implemented and evaluated in primary education. In this approach, measures are planned and implemented to remove or modify causes of work stress and evaluated in a controlled trial. Since this type of approach targets work stress risks at their source, it holds potential to sustainably decrease work stress. However, as Nielsen, Taris and Cox pointed out (Nielsen et al., 2010a), this type of approach can only be effective if the planned measures are appropriate to target the work stress risks, and if the approach is successfully implemented. To diminish the risks of selecting inappropriate effective measures and/or implementation failure, as compared to other work stress prevention approaches, the approach in our study is expanded in two ways.

First, a logic model of change is built as part of the risk assessment to facilitate the selection of appropriate measures. A logic model of change represents the pathways of the work stress prevention approach’s effects. By building a logic model of change, it is made explicit what behavioral change the measures should aim for per stakeholder involved, but also what determinants the measures should target, and thus, what change methods the measures should contain. Providing working groups with the rationale behind potential measures by providing a logic model of change may facilitate working groups to select and plan appropriate measures.

Second, during implementation, progression on outcomes, risk factors, target behaviors as well as implementation factors are real-time monitored and fed back to the working groups. Feeding back monthly progression on outcomes, risk factors, and target behaviors is assumed to contribute to goal pursuit and to motivate working groups to adjust action plans when needed. Feeding back monthly monitoring data on implementation factors (employee participation, communication, commitment, and readiness for change) provides working groups with the opportunity to take action to optimize implementation and reduce the risk of implementation failure. The working groups are in charge of translating the monitoring results into actions, measures, or interventions (e.g., Is more communication needed? When? In what form? To whom?). This provides opportunities for the working groups to experiment with actions to optimize implementation, resulting in active learning in the project, but also for the longer term.

Although we propose that these additions to the common work stress prevention approach could increase its’ potential success, there are some challenges to this approach as well that have to be taken into account. Work stress prevention approaches require effort from all members of the organization (Nielsen et al., 2010a), often in situations where demands already are high. Adding additional activities to this approach will even further increase the effort needed from participants.

First, the approach requires extra time and effort from employees within the intervention schools. Participating in monthly EMA measurements requires time and effort of participants, which already are confronted with high job demands. To reduce the risk of response loss, the monthly surveys are as short as possible and provide participants with feedback on their work stress levels within the app. This could work as an incentive to participate and could actually help in monitoring employees’ stress levels and take appropriate action.

Second, the approach requires extra time and effort of the working groups. Although the monthly reports on outcomes, risk factors, target behaviors, and implementation are aimed to facilitate the working groups during implementation and ultimately save time, the reports also require extra time and effort of the working group members to read and reflect on them. From earlier research (e.g., Bakhuys Roozeboom et al., 2020), it is known that lack of time or priority of different stakeholders (e.g., working group and management) are important barriers for implementation. For this reason, it is important for the working groups to find the right frequency of working group meetings (enough meetings to ensure commitment and priority, and not taking too much time). To facilitate this, the working groups can tailor the frequency of meetings to fit with their needs and work schedules. On the other hand, the regular feedback reports can also work as a cue for working group members to keep on prioritizing the project in daily working life.

There are some strengths and limitations in relation to the overall study design as well. A strength of the study is that effects are evaluated using a controlled trial design with 2 years of follow-up. This makes it possible to evaluate changes over time and draw conclusions on the effects of the approach. In addition, monthly monitoring of the implementation process provides quantitative data that can be used to draw a dynamic picture of the implementation process over time. Together with qualitative data (interviews and data logs), this offers a unique insight into how the implementation develops over time.

A limitation of the study design is that the control group is relatively larger than the intervention group. Based on the power calculation, the effect evaluation is not expected to be hindered by power issues, and efforts are made by the research team to encourage participants to take part in the measurements and optimize response, particularly in the intervention schools.

Although the work stress prevention approach will require efforts from the participants within the schools, we expect the benefits to outweigh the costs. Given the scarcity of teachers, the high prevalence of work stress, and the severe consequences, we believe that there is a great urgency to sustainably reduce work stress in this sector. We aim for our study to contribute to solvation of this important issue by developing a new approach and providing more insights into work stress intervention research in primary education.

## Ethics Statement

The study protocol is reviewed by the Medical Ethics Review Committee of VU University Medical Center and the committee concludes that the Medical Research Involving Human Subjects Act (WMO) does not apply to the study and that an official approval of this study by the committee is not required. The participants provided their written informed consent to participate in this study.

## Author Contributions

MB conducted the study and was responsible for data collection and drafting the article. RS, NW, IN, and CB provided intellectual input. All authors provided comments on the draft versions and have read and approved the final version of the manuscript.

## Funding

The development and implementation of work stress prevention approach are funded by the school cooperations Stip and Cordeo, and TNO. The writing of the article was funded by TNO and financially supported by the Ministry of Social Affairs and Employment in Netherlands. No grants were received for the research.

## Conflict of Interest

The authors declare that the research was conducted in the absence of any commercial or financial relationships that could be construed as a potential conflict of interest.

## Publisher’s Note

All claims expressed in this article are solely those of the authors and do not necessarily represent those of their affiliated organizations, or those of the publisher, the editors and the reviewers. Any product that may be evaluated in this article, or claim that may be made by its manufacturer, is not guaranteed or endorsed by the publisher.

## Abbreviations

EMA: Ecological momentary assessment
ICC: Intraclass correlation coefficient
